# Transformation of multi-component ginkgolide into ginkgolide B by *Coprinus comatus*

**DOI:** 10.1186/s12896-015-0133-0

**Published:** 2015-03-14

**Authors:** HongXue Ding, ZhiCai Zhang, ShengNan Cao, Yin Xu, JianGuo Yu

**Affiliations:** School of Food Science and Biotechnology, Jiangsu University, Zhenjiang, Jiangsu 212013 P. R. China; Jiangsu Tongyuantang Bio-technology Co., Ltd., Taixing, Jiangsu 225403 P. R. China

**Keywords:** Ginkgolide B, Bio-transformation, *Coprinus comatus*, Proteomic analysis

## Abstract

**Background:**

As the strongest antagonist of the platelet activating factor, ginkgolide B (GB) possesses anti-ischemic, anti-oxidant and anti-convulsant properties, and it is used for the treatment of thrombosis in clinical practice. Till now, GB is usually obtained from extraction of *Ginkgo biloba* leaves through column chromatography with an extremely low yield and high cost, which can not meet clinical requirement. Therefore, it is urgent to find a new method to prepare GB.

**Results:**

In the current study, we studied the ability and mechanism to transform multi-component ginkgolide into GB by *Coprinus comatus* in order to enhance the GB yield. Except for ginkgolide A (GA) and GB, all the other ginkgolides in the extract were transformed by the strain. In the case of culture medium containing 20 g/L glucose, the transformation product was identified as 12% GA and 88% GB by high performance liquid chromatography-Mass spectrometry (HPLC-MS), two stage mass spectrometry (MS/MS) and nuclear magnetic resonance (NMR). Partial GA was also transformed into GB according to the yield (76%) and the content of GA in the raw ginkgolide (28.5%). Glucose was the key factor to transform ginkgolides. When glucose concentration in medium was higher than 40 g/L, all ginkgolides were transformed into the GB. Proteomic analysis showed that *C. comatus* transformed ginkgolide into GB by producing 5 aldo/keto reductases and catalases, and enhancing the metabolism of glucose, including Embden-Meyerhof pathway (EMP), hexose monophophate pathway (HMP) and tricarboxylic acid (TCA).

**Conclusions:**

*C. comatus* could transform ginkgolides into GB when the medium contained 40 g/L glucose. When the strain transformed ginkgolides, the glucose metabolism was enhanced and the strain synthesized more aldo/keto reductases and catalases. Our current study laid the groundwork for industrial production of GB.

## Background

**Figure 1:**
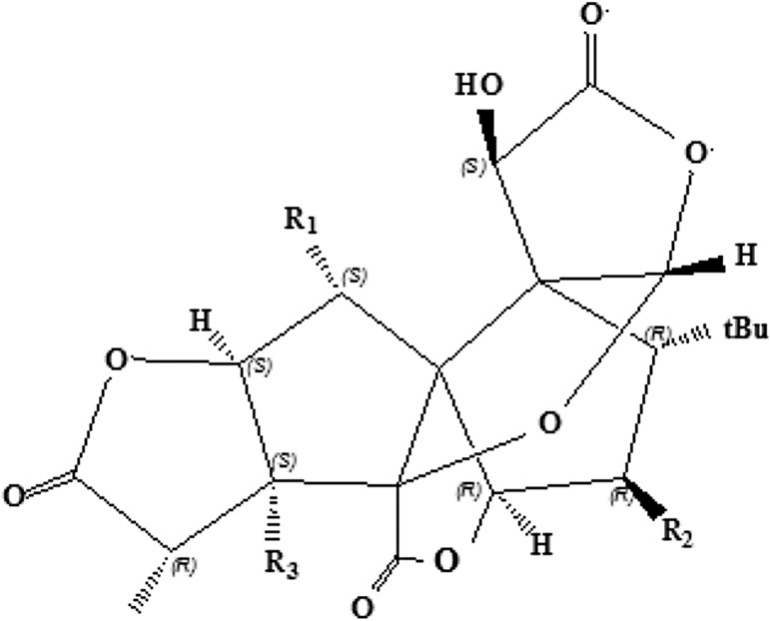
**The structure of ginkgolide (G).** GA: R_1_ = R_2_ = H, R_3_ = OH; GB: R_1_ = R_3_ = OH, R_2_ = H; GC: R_1_ = R_2_ = R_3_ = OH; GJ: R_1_ = H, R_2_ = R_3_ = OH; GM: R_1_ = R_2_ = OH, R_3_ = H.

The extracts of *Ginkgo biloba* leaves (EGB) possess anti-ischemic [1-3], anti-oxidant [4-6] and anti-convulsant properties [7]. A great deal of evidence supports that the bioactive components of EGB, including the terpene trilactones (TTLs) and flavonoids, have significant therapeutic effects on age-related physical and mental deterioration as well as cerebral vascular insufficiency, such as Alzheimer’s and cardiovascular diseases [8-10]. TTLs consist of ginkgolide A (GA), ginkgolide B (GB), ginkgolide C (GC), ginkgolide J (GJ) (Figure 1) and bilobalide (BB) [11]. As the main bioactive constituents of EGB, TTLs impart broad spectrum of pharmacological activities to plants.

Among TTLs, GB has many pharmacological functions, including anti-inflammatory, anti-tumor and ischemia-reperfusion protection effects, and it has long been used to treat central nervous system’s diseases, such as degenerative dementia and neurosensory disorders [12]. GB is the strongest antagonist of the platelet activating factor and used for the treatment of thrombosis in clinical practice [13,14]. However, the chemosynthesis of GB is very difficult due to its unique structure, and the yield of GB from EGB by chromatographic separation remains very low. Different methods have been reported to improve GB preparation [15-19], but they are all difficult to apply into industrial production.

Bio-transformation is an efficient approach to modify structures of complex natural products [20,21]. Bio-transformation reaction has many advantages, such as high regio- and stereo-selectivity; and mild reaction conditions compared with chemical synthetic methods [21,22]. Till now, many novel chemicals have been obtained from the monomers of natural compounds by this transformation approach, including flavonoids [23], terpenoides [24], saponins [25] and alkanes [26]. Few studies reported that one type of natural compound is synchronously transformed by one specific strain. In our previous studies, we proved that the edible and medicinal fungi can enhance biological functions of herbs through bio-transformation [27-30]. However, very limited information is available on how these edible and medicinal fungi transform the components of herbs. In order to explore its underlying mechanism, we compared the different edible and medicinal fungi, including *Lentinus edodes*, *Agaricus bisporus*, *Coprinus comatus*, *Ganoderma lucidum*, *Hericium erinaceus*, *Pleurotus ostreatus*, *Pleurotus eryngii*, *Boletus edulis* and *Cordyceps sinensis*, and components of herbs, including flavonoids, terpenoids, alkaloids, anthraquinones and so on. We found that many edible and medicinal could transform natural components of herbs; especially *C. comatus* could transform ginkgolide.

In the present study, we separated the transformation products of total ginkgolide by *Coprinus comatus* UJS03 through extraction and recrystallization. Moreover, we analyzed the structure of its individual components by comparing the retention time of high performance liquid chromatography (HPLC), nuclear magnetic resonance (NMR), infra-red spectrum (IR spectrum) and mass spectrum (MS) with the corresponding spectra of their standard substances. Taken together, this is the first study reporting that one type of compounds was synchronously converted into a simple compound by one specific strain through bio-transformation.

## Methods

### Microorganisms, media and culture conditions

A culture of *C. comatus* UJS03 was kindly provided by Anhui Bioscience & Technology Co., Ltd. The stock culture was maintained on potato dextrose agar (PDA) slants at 25°C for 9 days and then stored at 4°C. Total ginkgolide, composed of GA 28.50%, GB 21.26%, GC 24.15%, GM 8.69%, GJ 11.12% and BB 6.28%, was purchased from Xuzhou Kangruilai Bio-production Co., Ltd. (Xuzhou, China). The standards of GA and GB were obtained from Chinese Food & Drug Inspection Institute.

The medium applied in the transformation experiments was prepared using distilled water and composed of following components: glucose 20 g/L, corn powder 15 g/L, wheat bran 5 g/L, KH_2_PO_4_ 2 g/L, MgSO_4_•7H_2_O 3 g/L, yeast extract 2 g/L and corn steep powder 5 g/L. A 250-mL flask containing 60 mL medium was inoculated by punching out 5 mm of the agar slant culture with a self-designed cutter. The seed culture was grown in a rotary shaker at 150 rpm and 25°C for 7 days. Subsequently, 10 mL seed culture was inoculated into other 250-mL flasks containing similar medium and 0.5% total ginkgolide, which were incubated under the same conditions for 7 days. The broth was used to extract ginkgolide. The biomass was used in proteomics analysis, and the biomass without ginkgolide supplementation served as the control in proteomic analysis.

### Isolation and purification of transformed ginkgolide products

The broth and mycelium were separated through filtration after 168 h of incubation. The mycelia was dried at 80°C until a constant weight and then extracted at 80°C using 10 volumes of ethanol (v/w) for three times. The obtained extracts as well as the filtrate were combined and dried in rotary-evaporator under vacuum. The dried extract was dissolved using 10 volumes of ethyl acetate (v/w) at the room temperature. The resulting solution was filtrated, and the filtrate was washed by 3% NaHCO_3_ for three times and then concentrated to dry. The residual was dissolved with 10 volumes of ethanol. The solution was precipitated for 6 h by adding three volumes of distilled water (v/v). The precipitate was dissolved in small amount of ethanol and recrystallized at -20°C for 6 h. The crystal was obtained through filtration, washed with distilled water and then dried under the infrared ray.

### Analysis of GA and GB contents

#### Analysis of HPLC

HPLC analysis was performed on a Water Xbridge BEH C-18 column (2.5 μm, 3.0 × 100 mm) using high pressure binary pump, Waters 996 diode array detector and Agilent 1200 Series auto-sampler. The elution was obtained with a gradient of methanol (70%) and water (30%) at a flow rate of 0.2 mL/min. The HPLC system was coupled with a Agilent three-stage quadrupole mass spectrometry 6400 ion trap mass spectrometer (USA) operated in the mode of full-scan negative ion electrospray ionization (ESI) (m/z 500-800). MS parameters were optimized to maximum sensitivity as follows: ESI, negative polarity ionization; spray voltages: 4.0 kV; heating capillary temperature: 275°C; sheath gas (N_2_) flow: 30 Arb; Aux/sweep gas (N_2_); flow rate: 50 Arb.

#### Analysis of NMR and mass spectra

The ^1^H and ^13^C NMR spectra were determined using the Bruker Avance III 500 spectrometer instrument. High-resolution mass spectra were recorded on an LCQ-DECA ion trap spectrometer (ThermoFisher, USA) using ESI in negative polarity ionization.

### Comparative proteomic analysis of *C. comatus* in response to ginkgolide challenge

#### Frozen protein extraction

Frozen *C. comatus* mycelia (5 g) cultured in above-mentioned medium with or without ginkgolide supplementation were finely powdered in liquid nitrogen and homogenized in ice-cold homogenizing buffer, containing 1.05 mol L^−1^ sucrose, 60 mmol L^−1^ Tris, 10 mmol L^−1^ ethylene glycol bis (2-aminoethyl) tetraacetic acid (EGTA), 1 mmol L^−1^ phenylmethanesulfonyl fluoride (PMSF), 1 mmol L^−1^ dithiothreitol (DTT) and 1% (v/v) Triton X-100, pH 8.35. The homogenate was centrifuged at 5,000 × g for 15 min at room temperature, and the supernatant was collected and centrifuged again. The supernatant was the protein extracts and stored at -80°C for isoelectric focusing (IEF). The concentrations of the protein extracts were determined by the Bradford method [31].

#### Two-dimensional electrophoresis (DE)

The sample aliquots containing 1,500 μg proteins were mixed with fresh rehydration buffer, consisting of 9 mol/L urea, 4% 3-[(3-Cholanidopropyl)dimethylammonio]-1-propanesulfonate, 1% DTT, 1% immobilized pH gradient (IPG buffer) (GE Healthcare) and trace amount of bromophenol blue, to a total volume of 450 μL. Samples were placed into the strip holder (GE Healthcare, 24 cm, pH = 3-10, NL) after the drystrips were obtained from -20°C freezer and placed at room temperature for 10 min. IEF was performed on an IPGhor IEF System (GE Healthcare). Parameters for IEF were set as follows: temperature: 20°C; 50 μA per strip; rehydration at 50 V for 12 h (step), 500 V for 1 h (step), 1,000 V for 1 h (step), 10,000 V for 1 h (gradient), 10,000 V for 11 h (step).

The strips were gotten from the strip holder, and the redundant oil and protein solution were absorbed by filter paper. The strips were incubated in equilibration buffer 1, containing 6 mol/L urea, 30% glycerol, 2% sodium dodecyl sulphate (SDS), 50 mM Tris-HCl (pH 8.8), 1% DTT and trace amount of bromophenol blue, for 15 min and then in equilibration buffer 2, containing 6 mol/L urea, 30% glycerol, 2% SDS, 50 mM Tris-HCl (pH 8.8), 2.5% Iodoacetamide and trace amount of bromophenol blue, for 15 min. The strip was then removed and rinsed with SDS electrophoresis buffer (25 mmol/L Tris, 192 mmol/L glycine, 0.1%SDS) for 10 sec. After melted, the sealing solution (25 mmol/L Tris, 192 mmol/L glycine, 0.1% SDS, 0.5% agarose) was added to the surface of the SDS-PAGE gel (12% SDS gel solution: 12% acrylamide, 0.32% N N’-methylenebisacrylamide, 0.375 mol/L pH 8.8, Tris-HCL, 0.1% SDS, 0.05% ammonium persulphate, 0.05% N,N,N’,N’-Tetramethylethyl –enediamine (TEMED)), and the strip was laid across the top of the gel to make sure that the strip could be flushed with the gel. After the sealing solution was solidified, the gel was then moved to the electrophoresis apparatus for electrophoresis with parameters as follows. The equipment was Ettan-DALT-Six system; the temperature was at 15°C; gel running time was 45 min at 100 V and then 6-8 h at 200 V.

#### Protein staining and analysis of 2-D gels

After the electrophoresis, the gel was stained with Coomassie brilliant blue (CBB) according to Candiano’s protocol [32]. All gel images were processed by three steps, spot detection, volumetric quantification and matching, using Pdquest 8.0 software. The differences in protein content between treatment and control groups were determined as the fold ratio. Spots of ≥2 fold or ≤ 0.5 fold thresholds with a *p* value of less than 0.05 were excised from the gels, washed with double-distilled water and then transferred to sterilized Eppendorf tubes.

#### In-gel protein digestion and identification by matrixassisted laser desorption/ionization time-of-flight (MALDITOF/TOF)

The different protein spots were destained with 25 mmol/L NH_4_HCO_3_ and 50% acetonitrile (ACN) at room temperature for 30 min, and then they were sequentially dehydrated in 50% acetonitrile for 30 min and 100% acetonitrile for 30 min. After removing 100% ACN, the proteins were digested with 0.02 μg/μL trypsin (Promega, USA) at 37°C overnight (about 16 h). After hydrolysis, the supernatants were transferred into another tube. The residual gel was extracted again with extraction solution (5% trifluoroacetic acid (TFA), 67% ACN) at 37°C for 30 min and then centrifuged for 5 min. The obtained supernatant was combined, completely dried and re-suspended in 5 μL 0.1% TFA. Subsequently, it was mixed with equal volume of matrix consisting of a saturated solution of α-cyano-4-hydroxy-trans-cinnamic acid in 50% ACN, 0.1% TFA. Then 1 μL mixture was spotted on a stainless steel sample target plate. Peptide MS and MS/MS were performed on an ABI 5800 MALDI-TOF/TOF Plus mass spectrometer (Applied Biosystems, Foster City, USA). Data were acquired in a positive MS reflector using a CalMix5 standard to calibrate the instrument (ABI4800 Calibration Mixture). Both the MS and MS/MS data were integrated and processed by using the GPS Explorer V3.6 software (Applied Biosystems, USA) with default parameters. Based on combined MS and MS/MS spectra, proteins were successfully identified based on 95% or higher confidence interval of their scores in the MASCOT V2.3 search engine (Matrix Science Ltd., London, U.K.) according to following parameters: NCB Inr-Funji database; trypsin as the digestion enzyme; one missed cleavage site; fixed modifications of Carbamidomethyl (C); partial modifications of Acetyl (Protein N-term), Deamidated (NQ), Dioxidation (W), Oxidation (M); 100 ppm for precursor ion tolerance and 0.5 Da for fragment ion tolerance.

## Results and discussion

### The yield and analysis of the components of transformation products

Through bio-transformation of the total ginkgolide by *C. comatus*, the yield of GB was 76% and much higher compared with those obtained using other extraction methods because the theoretical maximum GB yield of conventional methods could not be higher than the GB content in the raw materials (26.12%). The control culture of *C. comatus* without ginkgolide supplementation did not produce GB. The result showed that *C. comatus* could not synthesize GB from scratch. Therefore, it suggested that *C. comatus* could transform other ginkgolides into the GB. To verify our deduction, all components of the product were analyzed with HPLC-MS, MS-MS and NMR spectra, respectively.

### Analysis of HPLC-MS

**Figure 2:**
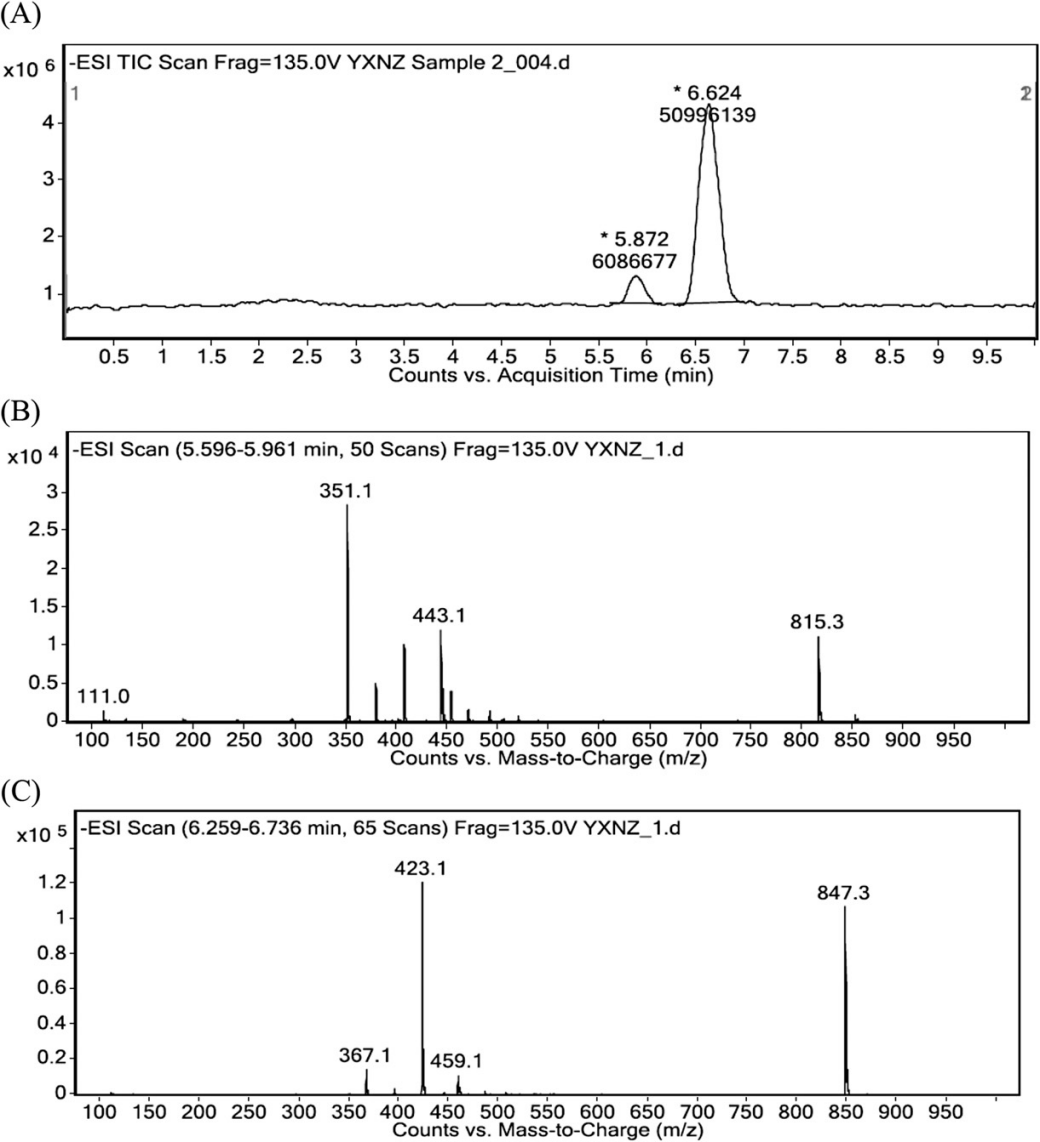
**HPLC-MS chromatograms of the transformation product. (A)** HPLC analysis of the product; **(B)** ESI-MS (-) MS spectrum of the retention time at 5.586 min; **(C)** ESI-MS (-) MS spectrum of the retention time at 6.387 min.

The LC-MS/MS method was first applied to analyze the components of bio-transformation product. Its total ion chromatograms (in Figure 2A) only exhibited two peaks of which retention times and molecular ion peaks at m/z were consistent with GA (5.745 min, 407, Figure 2B) and GB (6.463 min, 423, Figure 2C), respectively, and the peaks of GC, GM, GJ and BB were not detected. Therefore, these two peaks were deduced as the GA and GB, and other compounds were transformed or degraded by *C. comatus*. However, the production yield (76%) and total content of GA (28.50%) and GB (21.26%) in raw materials exhibited that the GC, GM and GJ were transformed into GA or GB if the two components in the production were GA and GB. Based on the external standard method and area of peaks, the content of GA and GB was 12% and 88%, respectively.

### Analysis of MS/MS

**Figure 3:**
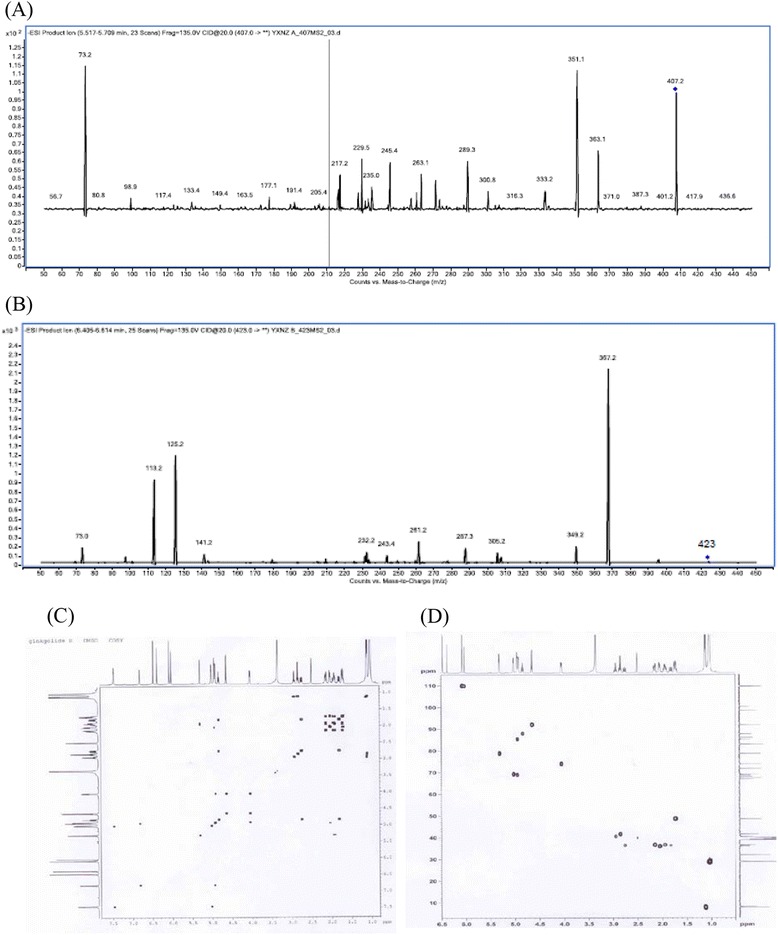
**MS/MS and NMR chromatograms of product. (A)** ESI-MS (-) MS spectrum of GA (m/z 408); **(B)** ESI-MS (-) MS spectrum of GB (m/z 423); **(C)** the 1H-1H COSY spectra; **(D)** the 1H-13C HSQC spectra.

In order to further identify these two peaks, MS/MS was used to compare the mass spectra of the product and standards of GA and GB. The results showed that in the negative mode, the two molecular ion peaks [M-H]^-^ of sample were at m/z 407 and 423 in a mass spectrometry (Figure 3A and B), and their fragment spectra at m/z 351 and 333 (Figure 3A) and 367, 345 (Figure 3B) were similar to those of the standard GA and GB, respectively. These results further proved that transformation products were GA and GB. In the spectra of MS/MS, the disappearance of molecular ion peak at m/z 325 demonstrated that BB was degraded or transformed by *C. comatus*. No detection of other molecular ion peaks also exhibited that the product did not contain other impurities other than GA and GB.

### Analysis of the ^1^H, ^13^C NMR and the heteronuclear singular quantum correlation (HSQC) spectra

Analysis of the ^1^H, ^13^C NMR and the HSQC spectra (Figure 3C and D) showed the ^1^H-^1^H COSY and ^1^H-^13^C HSQC spectra of the product. The signal assignments for ^1^H NMR and ^13^C NMR spectrometry in Figure 3C and D were shown in Tables 1 and 2. Table 2 reveals that two components of the product contained the same number of carbons and possessed the similar structure. The disappearance of 7-OH group (in Table 1) exhibited that the product did not contain GC, GM and GJ, which were transformed by *C. comatu*. The ^1^H, ^13^C NMR, and the COSY, HSQC spectra of two components in the product were consistent with the spectra of the standard GA and GB (Figure 3C and D). The significant difference of main carbon’s chemical shift between GA and GB was found between C-1, C-5 and C-9 of two components. The chemical shift of hydrogen atom of the corresponding carbon atoms exhibited significant differences (Table 1). These differences were caused by the hydroxyl group in C-1 of GB. The content of GA and GB was 12% and 88%, respectively, based on the integral quantification of 10-hydroxyl groups in the ^1^H-^1^H COSY spectra. Moreover, our data showed that no other ^1^H NMR and ^13^C spectrometry belonged to GA and GB in the ^1^H-^1^H COSY and ^1^H-^13^C HSQC spectra, confirming that no other impurities existed in the production.

**Table 1:** **Assignment of the** ^**1**^ **H-** ^**1**^ **H correlation signals found in the two-dimension COSY spectra of the transformed production**

**Chemical shift (ppm)**	**J (Hz)**	**Number of proton**	**Assignment** ^**a**^	^**1**^ **H-** ^**1**^ **H COSY**
7.46	d/5.0	1	10-OH (B)	H10 (B)
6.82	d/5.2	0.15	10-OH (A)	H10 (A)
6.47	s	1	3-OH (B)	
6.38	s	0.15	3-OH (A)	
6.07	s	1	H12 (B)	
6.02	s	0.15	H12 (A)	
5.30	d/4.0	1	H6 (B)	H7a, H7b (B)
5.02	d/5.0	1	H10 (B)	10-OH (B)
4.92	m	1	1-OH (B)	H1 (B)
		1.14	H6, H10 (A)	H7, 10-OH (A)
4.83	dd/7.2,8.0	0.15	H2 (A)	H1a, H1b (A)
4.64	d/7.4	1	H2 (B)	H1 (B)
4.04	dd/3.0,7.4	1	H1 (B)	H2, 1-OH (B)
2.93	q/7.2	0.15	H14 (A)	H16 (A)
2.83	q/7.1	1	H14 (B)	H16 (B)
2.75	dd/7.2,15.2	0.15	H1a (A)	H1b, H2 (A)
2.15	m	1	H7a (B)	H7b, H8 (B)
2.02	m	1.14	H7 (A)	H8 (A)
1.96	m	1	H7b (B)	H6, H7a, H8 (B)
1.81	dd/8.0,15.2	0.15	H1b (A)	H1a, H2 (A)
1.72	m	1	H8 (B)	H7a, H7b (B)
		0.15	H8 (A)	H7 (A)
1.11	m	3	H16 (B)	H14 (B)
		0.51	H16 (A)	H14 (A)
1.03	s	9	H18, H19, H20 (B)	
		1.53	H18, H19, H20 (A)	

^a^(A): H assigned ginkgolide A; (B): H assigned ginkgolide B.

**Table 2:** **Assignment of the** ^**1**^ **H -** ^**13**^ **C correlation signals found in the two-dimension HSQC and HMBC of transformed production**

**Chemical shift (ppm)**	**DEPT**	**Numbeer of Carbon**	**Assignment** ^**a**^	**HSQC**	**HMBC**
176.60	C	0.15	C15 (A)		H2, H14, H16 (A)
176.39	C	1	C15 (B)		H2, H14, H16 (B)
174.34	C	0.15	C11 (A)		H10, H12 (A)
173.95	C	1	C11 (B)		H10, H12 (B)
170.80	C	0.15	C13 (B)		
170.28	C	1	C13 (B)		H6 (B)
109.61	CH	1	C12 (B)	H12 (B)	H8, 3-OH (B)
109.51	CH	0.15	C12 (A)		H8 (A)
100.26	C	0.15	C4 (A)		H1a, H12, 3-OH (A)
98.45	C	1	C4 (B)		H12, H14, 3-OH (B)
91.81	CH	1	C2 (B)	H2 (B)	H1, H3 (B)
87.70	CH	0.15	C2 (A)	H2 (A)	H1b (A)
86.08	CH	0.15	C6 (A)	H6 (A)	
85.14	C	0.15	C3 (A)		H1a, H1b (A)
82.90	C	1	C3 (B)		H2, H14, H16, 3-OH (B)
78.60	CH	1	C6 (B)	H6 (B)	H7 (B)
73.76	CH	1	C1 (B)	H1 (B)	H2, H16 (B)
71.69	C	1	C9 (B)		
69.05	CH	1	C10 (B)	H10 (B)	H8, 10-OH (B)
68.74	CH	0.15	C10 (A)	H10 (A)	
68.06	C	0.15	C9 (A)		
67.42	C	1	C5 (B)		H6, H7, H10, 10-OH (B)
66.83	C	0.15	C5 (A)		H1a, H1b (A)
48.56	CH	1	C8 (B)	H8 (B)	H7, H10, H12 (B)
					H18, H19, H20 (B)
48.53	CH	0.15	C8 (A)	H8 (A)	
41.52	CH	1	C14 (B)	H14 (B)	H2, H3, H16 (B)
40.44	CH	0.15	C14 (A)	H14 (A)	
36.59	CH2	1	C7 (B)	H7 (B)	H8 (B)
36.31	CH2	0.15	C7 (A)	H7 (A)	
35.94	CH2	0.15	C1 (A)	H1a, H1b (A)	
31.97	C	1	C17 (B)		H8, H18, H19, H20 (B)
31.95	C	0.15	C17 (A)		
28.87	CH3	3	C18, C19, C20 (B)	H18. H19, H20	H8 (B)
		0.51	C18, C19, C20 (A)	H18, H19, H20	H8 (A)
8.19	CH3	0.15	C16 (A)	H16 (A)	
7.85	CH3	1	C16 (B)	H16 (B)	H14 (B)

^a^(A): C assigned ginkgolide A; (B): C assigned ginkgolide B.

In summary, the product contained two components of GA and GB, and their contents were 12% and 88%, respectively. If GA in raw materials was not transformed to GB, and other ginkgolides were all transformed into GB, the theoretical maximum content should be 72.5% because the GA content in raw materials was 28.5% and other ginkgolides content in raw materials was less than the true content of production (88%). Based on the facts, it was believed that the partial GA was also transformed into GB or degraded.

### The mechanism of ginkgolide transformation into GB by *C. comatus*

**Figure 4:**
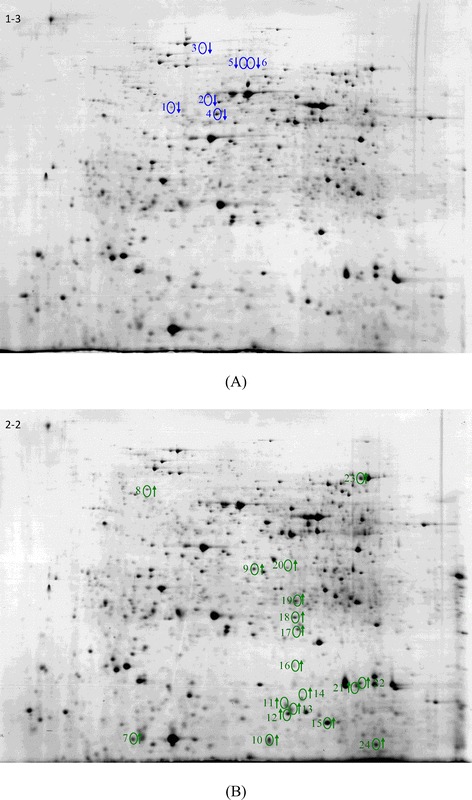
**The 2D-gel analysis of** ***C. comatus*** **proteins extracted from mycelia. (A)** grown in media not supplied ginkgolide; **(B)** grown in media supplied ginkgolide.

To disclose the mechanism that *C. comatus* transformed ginkgolides, the proteins difference of *C. comatus* biomass between transforming and un-transforming ginkgolide was analyzed. Comparative analysis of 2-DE gels between the medium with and without ginkgolide supplementation showed that the 24 proteins spots were differently expressed in response of the ginkgolide challenge (Figure 4A and B). Among these different proteins, six proteins were down-regulated and 18 proteins were up-regulated. The down-regulated proteins included chaperonin GroL (3711), succinate-CoA ligase (2710), NADP-binding protein (4701), hypothetical protein HETIRDRAFT-413641 (4816), V-type ATPase (3909) and an unknown protein (4817). The up-regulated proteins included 6-phosphogluconate dehydrogenase (6108), glyceraldehyde 3-phosphate dehydrogenase (6302), glucose-6-phosphate isomerase (8002), malate dehydrogenase (6303), 5 aldo/keto reductases (6201, 5119, 5113, 6103, 1007), catalase (7820), UDP-glucose 4-epimerase (6402), cobalamin-independent methionine synthase (7204), acetamidase/formamidase (2803), cyclophilin (7203), 3 hypothetical protein (5513, 4513, 5114) and an unknown protein (5008).

It is known that glucose-6-phosphate isomerase and glyceraldehyde 3-phosphate dehydrogenase are the rate limiting enzymes of Embden-Meyerhof pathway (EMP), 6-phosphogluconate dehydrogenase is the rate limiting enzyme of hexose monophophate pathway (HMP), and malate dehydrogenase is the rate limiting enzyme of tricarboxylic acid (TCA) cycle. Our data suggested that HMP, EMP and TCA involved into glucose metabolism were enhanced in the response of ginkgolide challenge. In the HMP, 1 mol glucose-6-phosphate is converted into the 6-phosphogluconate and ribulose-5-phsphate by two steps of dehydrogenation reaction, which produces 2 mol reduced form of nicotinamide-adenine dinucleotide phosphate (NADPH) used as the reducing powder (NADPH). Moreover, 1 mol glucose is converted into 1 mol glucose 6-phosphate, 1 mol fructose 6-phosphate is converted into 1 mol fructose-1,6-biphosphate by two steps of phosphorylation of EMP and consume 2 adenosine-triphosphate (ATP). However, 1 mol fructose-1,6-biphosphate can form glyceraldehydes 3-phosphate and dihydroxyacetone phosphate that can synthesize 4 ATP and 2 NADH by EMP. Therefore, 1 mol glucose can produce 2 mol ATP, 2 mol NADH and 2 mol pyruvate by EMP. In addition, 1 mol pyruvate is oxidized into CO_2_ and H_2_O by TCA pathway, creating 4 mol reduced nicotinamide adenine dinucleotide (NADH), 1 mol reduced flavin adenine dinucleotide (FADH) and 1 mol GTP, which is equal to 15 ATPs. Therefore, 1 mol glucose can produce 38 mol ATP by EMP and TCA cycle [33]. The increased activities of these key enzymes, including glucose-6-phosphate isomerase, glyceraldehyde 3-phosphate dehydrogenase of EMP, 6-phosphogluconate dehydrogenase of HMP and malate dehydrogenase of TCA, suggested that the metabolism of EMP, HMP and TCA was enhanced to provide NADPH and ATP to transform multi-component ginkolide.

UDP-glucose 4-epimerase catalyzes a freely reversible reaction between UDP-galactose and UDP-glucose, and it facilitates the utilization of galactose in the wheat bran of the medium. Cobalamin-independent methionine synthase catalyzes the synthesis of methionine by transferring a methyl group from methyltetrahydrofolate to homocysteine. Amidases are enzymes that catalyze the hydrolysis of amide compounds to their corresponding carboxylic acid and ammonia [34]. Formamidase (KFase) (EC 3.5.1.9) is a key enzyme in the metabolic sequence of tryptophan [33]. Their activity increase hinted that the catabolism of protein was enhanced to provide the ATP to transformation of ginkgolides. Succinate-CoA ligase, a mitochondrial matrix enzyme, catalyzes the reversible conversion of succinyl-COA and ADP or GDP to succinate and ATP or GTP, and at least the ADP-forming enzyme is part of the Krebs cycle. The down-regulation of succinate-CoA ligase suggested that more succinyl-COA was involved into TCA and TCA.

In contrast to the molecular structure of GC, GA, GB, GJ and GM, it is found that the difference between them depends on the different number and sites of the hydroxyl groups. GA contains one hydroxyl group on the R3 site, GB, GJ and GM all contain two hydroxyl groups on the R1 and R3 sites, 1 and R3, R2 and R3 sites, respectively. GC contains three hydroxyl groups on the R1, R2 and R3 sites. Therefore, if GA, GC, GM, and GJ are all transformed into GA, the H on the R1 site of GA and GJ and on the R3 site of GM are oxidized into OH, the OH on the R2 site of the GC, GJ and GM are reduced into H. Namely, there may be some oxidants and reductases in the internal mycelia. Catalase is an induced enzymes and converts hydrogen peroxide into harmless water and oxygen similar to superoxide dismutase to prevent free radical damage to the body. The up-regulation of catalase exhibited an increase of the H_2_O_2_content. H_2_O_2_ might oxidze H on the R3-site of GM and R1-site of GA and GJ into to OH. Aldo/keto reductases play a key role in reducing the OH on the R2 site of the GC, GJ and GM into the H group. The H of these reduction reactions all comes from the NADPH, whereas the energy comes from ATP. Therefore, if the amount of glucose in the medium of transformation experiment was increased, the slight amount of GA in production might be fully transformed to GB. To validate the deduction, the effect on transformation of *C. comatus* to ginkgolides was compared at different glucose concentrations, including 20 g/L, 30 g/L, 40 g/L, 50 g/L and 60 g/L, and we found that all ginkgolides, including ginkgolides A, were fully transformed into ginkgolides B when the glucose concentration in the medium was higher than 40 g/L.

Cyclophilins display the enzymatic activity of a peptidyl-prolyl isomerase (PPIase) [35,36], which catalyzes the cis to trans conversion of proline-containing peptides and facilitates protein folding. Protein folding *in vivo* is assisted by a set of proteins collectively known as molecular chaperones, which help successfully proteins fold to their native structures in the crowded environment of the living cells [37-39]. The up-regulation of cyclophilins and down-regulation of chaperones showed that the higher structure of some enzymes and proteins was changed to convert ginkgolides into GB.

V-ATPases from in all eukaryotic cells are a type of highly conserved protein and consist of more than 10 different subunits in two functional domains. A membrane-associated domain (V_0_) forms the proton channel, and a soluble catalytic domain (V_1_) is involved in ATP hydrolysis [40,41]. NADP-binding protein is a type of NADP dependent protein, and it controls the rate of oxidative phosphorylation reaction by involving in dehydrogenation reaction. The down-regulation of these enzymes and proteins exhibited that the ATP applied into proton channel was decreased to provide ATP and NADH to transform ginkgolide.

## Conclusions

We developed a method to transform ginkgolide into GB by strain *C. comatus*. The product yield reached 76%, and was much higher compared with conventional extraction. Analysis of LC-MS, MS/MS and NMR revealed that the transformation product included GB and GA when the medium only contained 20 g/L glucose, and their content was 88% and 12%, respectively. When the glucose concentration in medium was higher than 40 g/L, all ginkgolides were transformed into GB by *C. comatus*. Proteomic analysis further confirmed that the *C. comatus* transformed the ginkgolide, and the reduction power and energy for the bio-transformation were provided by NADPH and ATP, which were obtained in the process of glucose metabolism and synthesis. The method laid the found for biotransformation of active components in herbs.

## Abbreviations

EGB: Extracts of *Ginkgo biloba* leaves
TTLs: Terpene trilactones
GB: Ginkgolide B
GA: Ginkgolide A
GC: Ginkgolide C
GJ: Ginkgolide J
BB: Bilobalide
PDA: Potato dextrose agar
GTP: Guanosine-5′-triphosphate
ATP: Adenosine-triphosphate
NADH: Reduced nicotinamide adenine dinucleotide
FADH: Reduced flavin adenine dinucleotide
HPLC-MS: High performance liquid chromatography-Mass spectrometry
HPLC: Liquid chromatography
MS/MS: Two stage mass spectrometry
NMR: Nuclear magnetic resonance
IR spectrum: Infra-red spectrum
MS: Mass spectrum
IEF: Isoelectric focusing
COSY: Correlated spectroscopy
HSQC: Heteronuclear singular quantum correlation
ESI: Electrospray ionization
MALDITOF/TOF: Matrixassisted laser desorption/ionization time-of-flight
DE: Two-dimensional electrophoresis
EMP: Embden-Meyerhof pathway
HMP: Hexose monophophate pathway
TCA: Tricarboxylic acid
EGTA: Ethylene glycol bis (2-aminoethyl) tetraacetic acid
PMSF: Phenylmethanesulfonyl fluoride
DTT: Dithiothreitol
TEMED: N,N,N’,N’-Tetramethylethyl –enediamine
CBB: Coomassie brilliant blue
IPG buffer: Immobilized pH gradient
SDS: Sodium dodecyl sulphate
